# Large-Scale Imputation of KIR Copy Number and HLA Alleles in North American and European Psoriasis Case-Control Cohorts Reveals Association of Inhibitory KIR2DL2 With Psoriasis

**DOI:** 10.3389/fimmu.2021.684326

**Published:** 2021-06-11

**Authors:** Richard Ahn, Damjan Vukcevic, Allan Motyer, Joanne Nititham, David McG. Squire, Jill A. Hollenbach, Paul J. Norman, Eva Ellinghaus, Rajan P. Nair, Lam C. Tsoi, Jorge Oksenberg, John Foerster, Wolfgang Lieb, Stephan Weidinger, Andre Franke, James T. Elder, Eric Jorgenson, Stephen Leslie, Wilson Liao

**Affiliations:** ^1^ Department of Microbiology, Immunology, and Molecular Genetics, University of California, Los Angeles, Los Angeles, CA, United States; ^2^ Institute for Quantitative and Computational Biosciences, University of California, Los Angeles, Los Angeles, CA, United States; ^3^ Melbourne Integrative Genomics, The University of Melbourne, Parkville, VIC, Australia; ^4^ School of Mathematics and Statistics, The University of Melbourne, Parkville, VIC, Australia; ^5^ Clinical Epidemiology and Biostatistics Unit, Murdoch Children’s Research Institute, Parkville, VIC, Australia; ^6^ Department of Dermatology, University of California, San Francisco, San Francisco, CA, United States; ^7^ Department of Neurology, Weill Institute for Neurosciences, University of California, San Francisco, San Francisco, CA, United States; ^8^ Division of Personalized Medicine, Department of Immunology and Microbiology, University of Colorado, San Francisco, CA, United States; ^9^ Institute of Clinical Molecular Biology, Christian-Albrechts-University of Kiel, Kiel, Germany; ^10^ Department of Dermatology, University of Michigan, Ann Arbor, MI, United States; ^11^ Department of Biostatistics, Center for Statistical Genetics, University of Michigan, Ann Arbor, MI, United States; ^12^ Department of Computational Medicine & Bioinformatics, University of Michigan, Ann Arbor, MI, United States; ^13^ College of Medicine, Dentistry, and Nursing, University of Dundee, Dundee, United Kingdom; ^14^ Institute of Epidemiology, Christian-Albrechts-University of Kiel, Kiel, Germany; ^15^ Department of Dermatology, University Hospital Schleswig Holstein, Kiel, Germany; ^16^ Ann Arbor Veterans Affairs Hospital, Dermatology, Ann Arbor, MI, United States; ^17^ Division of Research, Kaiser Permanente, Oakland, CA, United States; ^18^ School of Biosciences, The University of Melbourne, Parkville, VIC, Australia

**Keywords:** psoriasis, KIR, HLA, imputation, genetics, autoimmunity

## Abstract

Killer cell immunoglobulin-like receptors (KIR) regulate immune responses in NK and CD8+ T cells *via* interaction with HLA ligands. KIR genes, including KIR2DS1, KIR3DL1, and KIR3DS1 have previously been implicated in psoriasis susceptibility. However, these previous studies were constrained to small sample sizes, in part due to the time and expense required for direct genotyping of KIR genes. Here, we implemented KIR*IMP to impute KIR copy number from single-nucleotide polymorphisms (SNPs) on chromosome 19 in the discovery cohort (n=11,912) from the PAGE consortium, University of California San Francisco, and the University of Dundee, and in a replication cohort (n=66,357) from Kaiser Permanente Northern California. Stratified multivariate logistic regression that accounted for patient ancestry and high-risk HLA alleles revealed that KIR2DL2 copy number was significantly associated with psoriasis in the discovery cohort (p ≤ 0.05). The KIR2DL2 copy number association was replicated in the Kaiser Permanente replication cohort. This is the first reported association of KIR2DL2 copy number with psoriasis and highlights the importance of KIR genetics in the pathogenesis of psoriasis.

## Introduction

Psoriasis is an inflammatory, immune-mediated skin disease affecting approximately 3% of the US population (1). Psoriasis has been associated with numerous systemic comorbidities including psoriatic arthritis, atherosclerosis, metabolic syndrome, type 2 diabetes, non-alcoholic fatty liver disease, and inflammatory bowel disease (2, 3). Psoriasis is known to be a complex heritable disease with over 64 genetic loci identified by genome-wide association studies in European and Asian cohorts (4, 5). The locus demonstrating the strongest effect on risk is the MHC region, with several alleles of *HLA-C* and *HLA-B* conferring a 2-4-fold increased risk of psoriasis (6). There are multiple biological mechanisms by which psoriasis-associated HLA alleles can increase disease susceptibility. We have previously shown (7), and other groups have confirmed (8), that there are canonical amino acid residues within the peptide binding groove of HLA-C and HLA-B that strongly associate with psoriasis, suggesting that HLA alleles with these residues may bind psoriasis autoantigens, several of which have been described (9, 10). HLA-B allotypes also bind to regulatory leukocyte immunoglobulin-like receptors (LILRs) on antigen presenting cells with differential affinity, which we have also shown associates with psoriasis susceptibility (11). Alternatively, class I MHC molecules including HLA-C and HLA-B may serve as ligands for killer immunoglobulin-like receptors (KIR), which are expressed on both natural killer (NK) cells and CD8+ T cells.

KIRs comprise a number of activating and inhibitory receptors that help regulate NK and CD8+ T cell responses and have been implicated in a number of clinical conditions, including susceptibility to a number of autoimmune disorders, viral infections, spontaneous abortion, and certain cancers (12). KIRs are encoded by a cluster of genes located on chromosome 19q13.4 (13) and different KIR haplotypes vary in the presence or absence of individual KIR genes, leading to KIR copy number variation in individuals. Moreover, much like the genes for their corresponding HLA ligands, KIR genes are highly multi-allelic and not easily amenable to direct genotyping *via* inexpensive genome-wide association study (GWAS) SNP panels. As a result, KIR copy number typing or allelic typing typically necessitates costly Sanger or next-generation sequencing.

**Figure 1:**
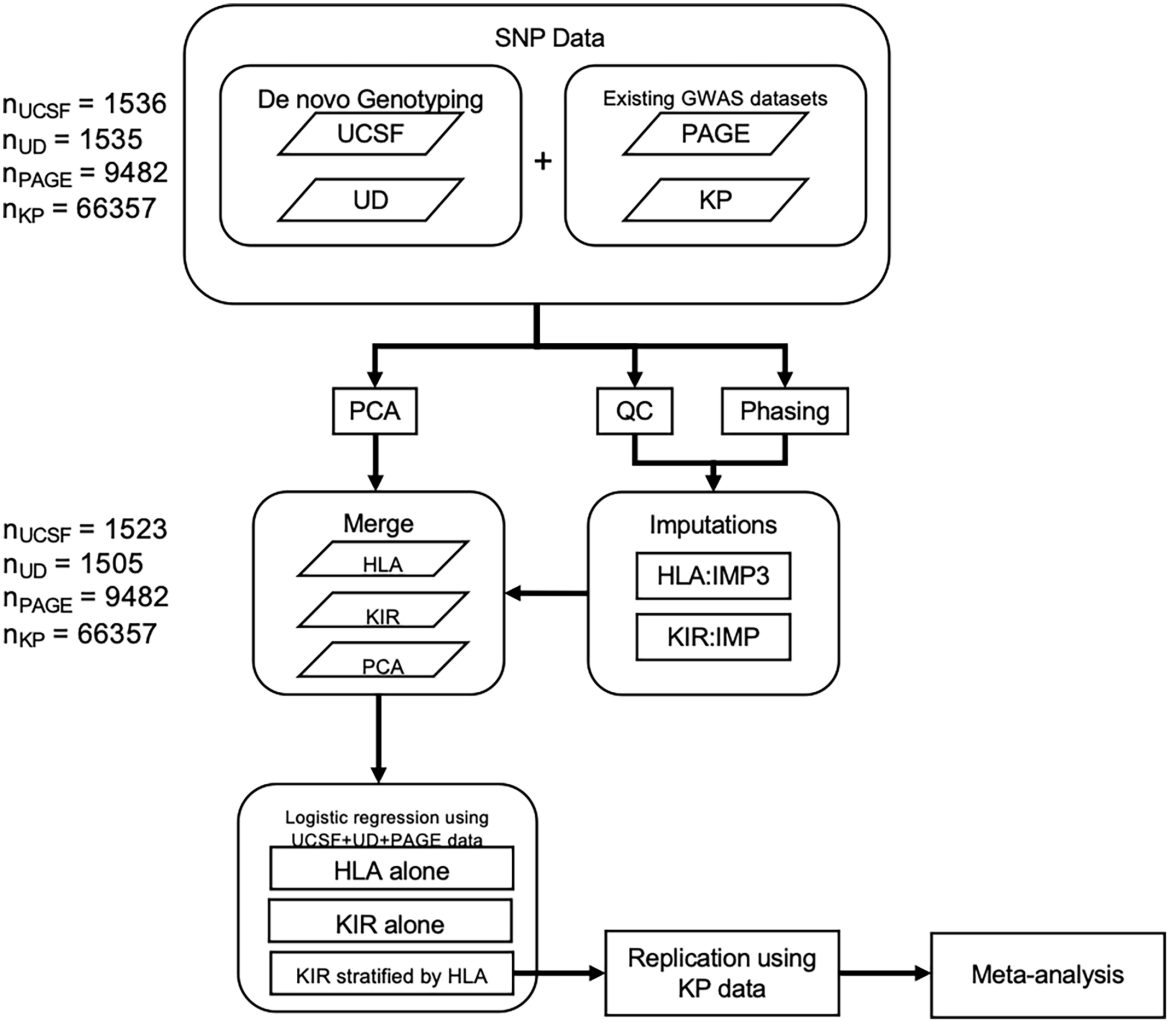
Methods flowchart. Flowchart outlining 1) the collection of SNP data from the PAGE consortium and KP as well as the genotyping of SNPs from the UCSF and University of Dundee datasets 2) QC, phasing, and imputation of HLA alleles and KIR CN 3) PCA using ancestry informative SNPs 4) merging of HLA, KIR, and PCA data 5) Association testing in the discovery cohort followed by replication and meta-analysis.

Although a number of prior genetic studies have suggested KIR associations with psoriasis, including *KIR2DS1* (12), *KIR3DL1* (14, 15), and *KIR3DS1* (7) these studies have been hampered by relative small sample sizes due to the expense of KIR sequencing. Over the last decade, efficient and accurate methods to impute HLA alleles from GWAS panels have been developed (16, 17) and have been leveraged to great success in several large-scale studies (7, 18, 19). More recently, we developed an accurate method to impute KIR copy number (KIR CN) using SNP data from GWAS arrays (20). In this present study we have imputed HLA alleles and KIR CN into large-scale psoriasis GWAS datasets totaling 10,066 cases and 68,203 controls (**Figure 1**). We used a discovery cohort (comprised of samples from UCSF, the University of Dundee, and the Population Architecture Using Genomics and Epidemiology (PAGE) consortium—referred to henceforth as PAGE+) to perform association testing of psoriasis with KIR CN alone, HLA ligands alone, and in a joint HLA-KIR CN model, and identified an association of psoriasis with *KIR2DL2* in HLA C1/C2 heterozygotes. The *KIR2DL2* CN association was replicated in an independent dataset, which consists of samples from Kaiser Permanente Northern California (KP). To our knowledge, this is the largest examination of HLA-KIR in any human disease to-date, and highlights the importance of KIR genetics in the pathogenesis of psoriasis.

## Methods

### Sample Collection and Genotyping

#### UCSF and University of Dundee

Psoriasis patient and healthy control saliva samples were obtained at UCSF (21) and the University of Dundee (22). Libraries were prepared from these saliva samples and genotyped on the Affymetrix UK Biobank platform. After genotyping, SNPs were called with Affymetrix Power Tools v 1.18.0 (Affymetrix Power Tools. http://www.affymetrix.com/partners_programs/programs/developer/tools/powertools.affx) using the recommended parameters in the Affymetrix Best Practices Workflow (Affymetrix, “Axiom^®^ Genotyping Solution Data Analysis Guide”, 2014. http://media.affymetrix.com/support/downloads/manuals/axiom_genotyping_solution_analysis_guide.pdf) (details in Supplementary Methods).

#### PAGE Consortium

We refer the reader to Tsoi et al. (23) for more details on the phenotyping or genotyping of the samples from the PAGE consortium.

### Principal Component Analysis

We implemented Eigenstrat (24) using 1,732 SNPs to perform principal components analysis (PCA) to compute the top 10 principal components (PCs) (details in Supplementary Methods).

### HLA Allele and KIR CN Imputation

After initial quality control and filtering of the genotype data from chromosomes 6 and 19, HLA-B and -C alleles were imputed using HLA*IMP:03 (16) while KIR CN imputation was performed using KIR*IMP (20). We used a posterior probability of 0.6 as a cutoff for poor quality HLA allele imputations. After this filtering step, the posterior probability of the remaining samples was > 0.95. As per recommendations from Motyer et al. (25), we did not apply a KIR CN call filter based on the posterior probability of the call.

We have also recently implemented a KIR typing algorithm that utilizes next-generation sequencing (NGS) data to call KIR types (26). 826 of the 954 samples we genotyped on the UK Biobank Affymetrix chip were also submitted for NGS sequencing (27) and had successful KIR typing. Across the 6 KIR genes we imputed, the concordance with the NGS-based KIR typing was 91.2%, demonstrating that KIR*IMP imputation is a reliable method for imputing KIR genes. The only KIR gene to have a concordance less than 90% was KIR2DL1 (76%).

### Statistical Modeling: Logistic Regression Models and Meta-Analysis

All data preparation and modeling was performed using R (28). The KIR CN only models included a factor variable (0, 1, or 2 copies) for each of the 6 KIR genes tested (KIR2DL1, KIR2DL2, KIR2DS1, KIR2DS4, KIR3DL1, and KIR3DS1). The HLA only models included a variable to indicate the presence of each of the 5 HLA ligand genotypes (HLA-C1, HLA-C2, HLA-Bw4, HLA-Bw4-80I, and HLA-Bw4-80T) as well as the presence of the known high-risk psoriasis alleles, HLA-C*06:02 and HLA-C*12:03 (8). To adjust for the effect of population stratification, we also included the top 10 principal components in each model that we tested as covariates. Fixed-effects meta-analysis was performed using the “meta” package in R by Schwarzer et al. (29).

In this study we examined the PAGE+ cohort (discovery), which consists of 7198 cases and 4714 controls of European descent from UCSF, the University of Dundee, and the PAGE consortium (23), and 2) the Kaiser Permanente (KP) cohort (replication), which consists of 2868 cases and 63489 controls of European from the Northern California Kaiser Permanente Health system (**Table 1**). We used HLA*IMP:03 (16) to perform imputation of class I HLA alleles (HLA-A, -B, and -C) to two field (4 digit) resolution and used KIR*IMP (20) to perform imputation of KIR copy number.

**Table 1:** Number of cases and controls from the discovery and replication cohorts.

Cohort	Cases	Controls
*Discovery cohort*		
UCSF	747	954
University of Dundee	763	676
PAGE-Germany	2178	543
PAGE-Estonia	856	1250
PAGE-North America	2654	1291
**Discovery total**	**7198**	**4714**
*Replication cohort*		
Kaiser Permanente	2868	63489
**Discovery and replication total**	**10066**	**68203**

## Results

### Association Testing of KIR Copy Number With Psoriasis

**Figure 2:**
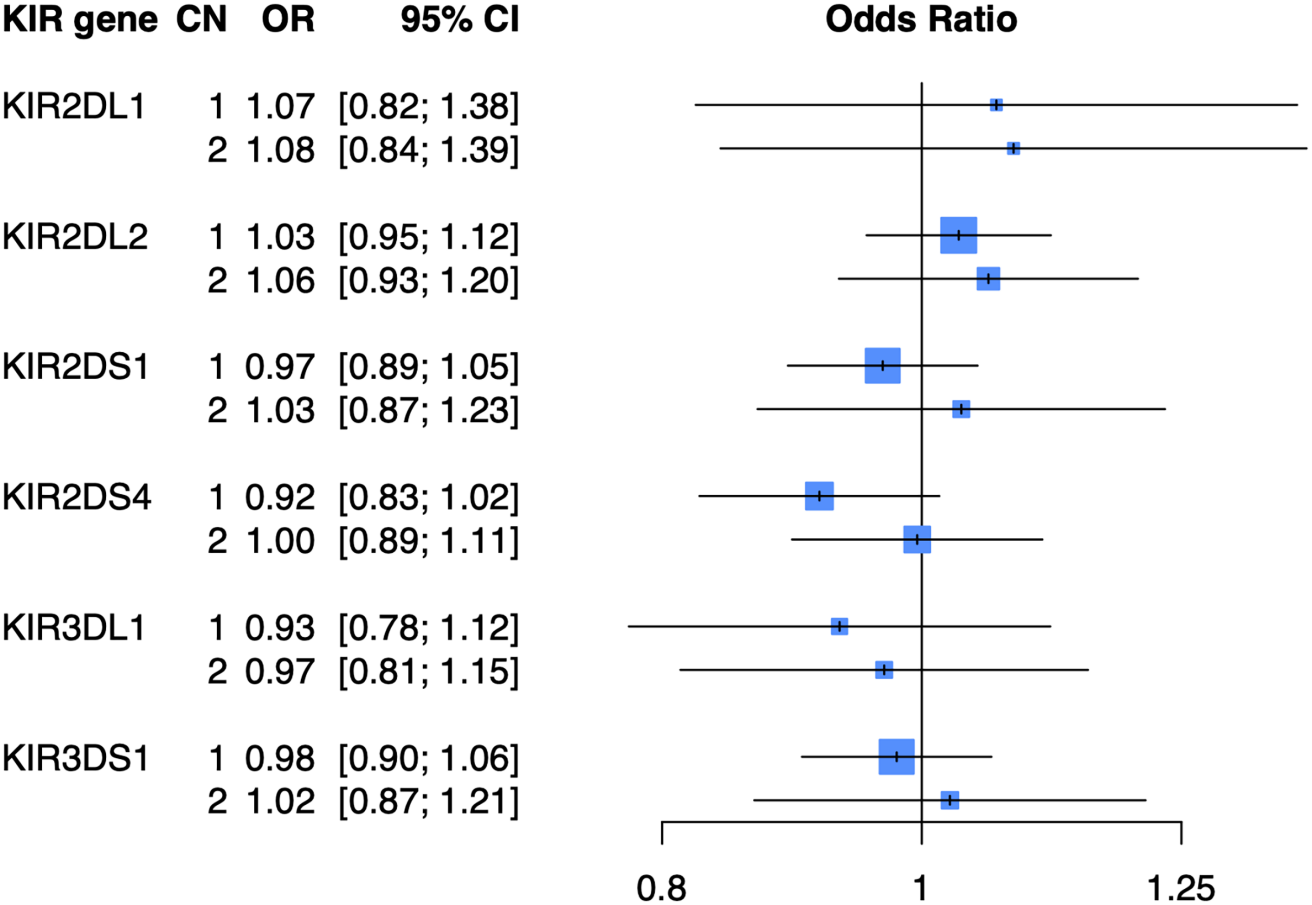
KIR copy number alone is not associated with psoriasis. Plot of ORs estimated from association testing of KIR CN with psoriasis after adjusting for the first 10 PCs.

To determine whether copy number of *KIR2DL1*, *KIR2DL2*, *KIR2DS1*, *KIR2DS4*, *KIR3DL1*, or *KIR3DS1* were associated with psoriasis independent of HLA ligands, we imputed the copy number (0, 1, or 2 copies) of these genes in the PAGE+ cohort of 7198 cases and 4714 controls and performed association testing using a general (0 *vs* 1 *vs* 2 copies) logistic regression model adjusted for the top 10 principal components (PCs) of ancestry. At a nominal p-value threshold of 0.05, we found that none of the six KIR genes tested were significantly associated with psoriasis (**Figure 2**). A dominant (0 *vs* 1 or 2 copies) logistic regression model adjusted for the top 10 PCs also yielded no significant associations with psoriasis (see **Supplementary Table 1** for full results).

### Association Testing of HLA Ligands With Psoriasis

**Figure 3:**
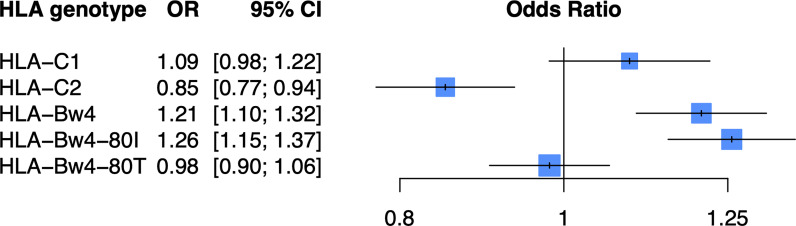
HLA-C2 and HLA-Bw4 are associated with psoriasis. Plot of ORs estimated from the dominant HLA model adjusted for HLA-C*06:02 and the top 10 PCs.

As KIR are known to bind the defined HLA ligands HLA-C1, HLA-C2, and HLA-Bw4 (**Table 2**), we examined whether these HLA ligands were associated with psoriasis in the PAGE+ cohort using a general and dominant logistic regression model adjusted for the top 10 PCs. We found that all three HLA ligands were significantly associated with psoriasis in the general and dominant model when not adjusting for any individual HLA alleles (**Supplementary Table 2**). However, after adjustment for the top psoriasis allele HLA-C*06:02, these associations were altered in the dominant model, with only HLA-C2 and HLA-Bw4 remaining significant. The HLA-Bw4 ligand may be further subdivided into HLA-Bw4-80I alleles and HLA-Bw4-80T alleles, which display differential binding affinity to *KIR3DL1* (44). We found that the association of HLA-Bw4 with psoriasis, after adjustment with HLA-C*06:02, was driven by HLA-Bw4-80I and not by HLA-Bw4-80T (**Figure 3**; see **Supplementary Table 2** for full results of models not adjusted for HLA-Cw6).

**Table 2:** Known interactions between KIR and HLA ligands.

KIR	HLA Ligand	Recent Citations
2DL1	HLA-C2	Winchester et al. (30), Yawata et al. (31), Campbell et al. (32), Parham et al. (33)
2DL2	HLA-C1 and HLA-C2	Martin et al. (34), Campbell et al. (32), Moesta et al. (35), Parham et al. (33), David et al. (36), Chandran et al. (37)
2DS1	HLA-C2	Luszczek et al. (38), Suzuki et al. (39), Holm et al. (40), Campbell et al. (32), Parham et al. (33)
2DS4	HLA-C1, HLA-C2	Hsu et al. (41), Maxwell et al. (42), Campbell et al. (32), Parham et al. (33)
3DL1	HLA-Bw4	Cella et al. (43), Campbell et al., (32), Parham et al. (33), Ahn et al. (14), Berinstein et al. (15)
3DS1	HLA-Bw4-80T	Campbell et al. (32), Parham et al. (33)

### KIR Copy Number Models Stratified on Their HLA Ligands Reveals Inhibitory KIR2DL2 Copy Number Is Associated With Psoriasis

**Figure 4:**
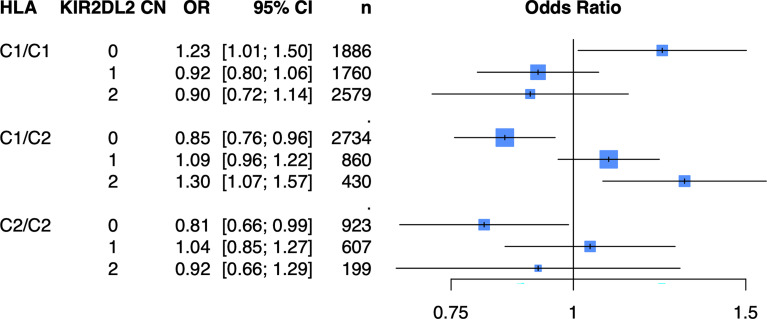
Having two copies of *KIR2DL2* is associated with psoriasis in the PAGE+ cohort when stratifying by HLA-C1/C2 after adjustment for HLA-C*06:02 and HLA-C*12:03. Plot of ORs from the *KIR2DL2* model adjusted for HLA-C*06:02 and HLA-C*12:03 as well as the top 10 PCs.

**Figure 5:**
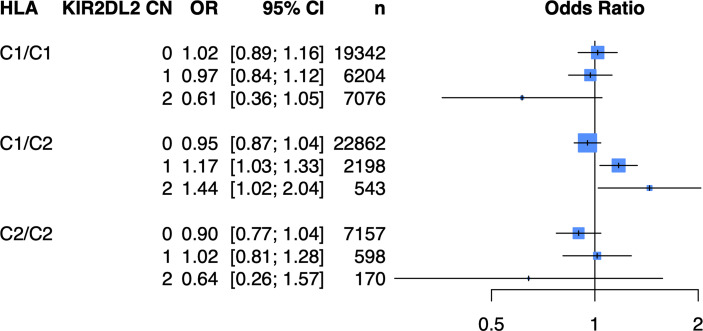
Having one or two copies of *KIR2DL2* is associated with psoriasis in the KP cohort when stratifying by HLA-C1/C2 after adjustment for HLA-C*06:02 and HLA-C*12:03. Plot of ORs from the *KIR2DL2* model adjusted for HLA-C*06:02 and HLA-C*12:03 as well as the top 10 PCs.

Because of the known biologic interaction between KIR and HLA ligands, we then tested joint models for KIR CN restricted to cases and controls who were positive for cognate HLA ligands (**Table 2**). Of the six KIRs and their respective HLA ligands, we found that having two copies of *KIR2DL2* (odds ratio (OR) = 1.25, 95% confidence interval (CI) [1.05 – 1.50]) in cases and controls who were HLA-C1/C2 heterozygous, was significantly associated with psoriasis in the PAGE+ cohort. *KIR2DL2* is known to biologically bind HLA-C1 and weakly interact with HLA-C2 (35). Sensitivity testing revealed that *KIR2DL2* remained associated with psoriasis even after including HLA-C*06:02 and HLA-C*12:03 alleles as covariates in the model, two individual HLA alleles that have been previously associated with psoriasis (8) (**Figure 4**; see **Supplementary Table 3** for full results). To confirm this result in an independent dataset, we tested the *KIR2DL2* CN model in HLA-C1/C2 heterozygotes and adjusted for HLA-C*06:02 and HLA-C*12:03 in the KP dataset and found a significant result (*KIR2DL2* CN = 1: OR = 1.17, CI [1.03; 1.33]; *KIR2DL2* CN = 2: OR = 1.44, CI [1.02; 2.04]) (**Figure 5**).

### Meta-Analysis of KIR2DL2 in the PAGE and KP Cohorts

**Figure 6:**
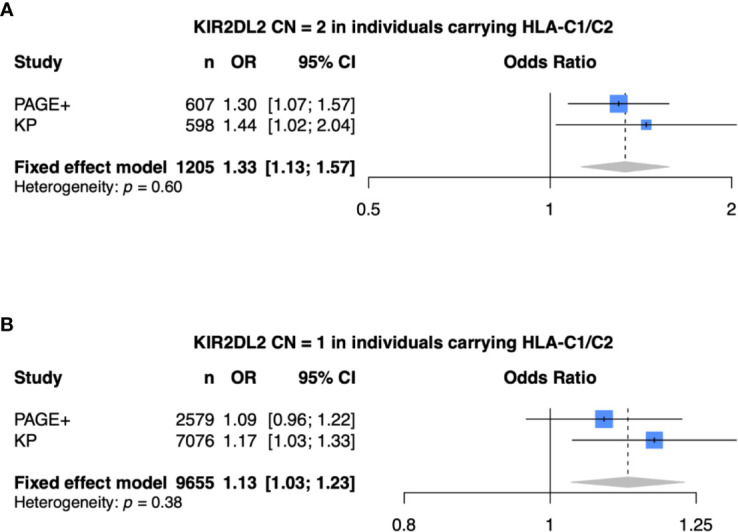
Meta-analysis shows *KIR2DL2* is associated with psoriasis. **(A)** Meta-analysis of model with 2 copies of KIR2DL2, stratified on HLA-C1/C2, and adjusted for HLA-C*0602, HLA-C*12:03, and the first 10 principal components (PCs). **(B)** Meta-analysis of model with 1 copy of KIR2DL2, stratified on HLA-C1/C2, and adjusted for HLA-C*0602, HLA-C*12:03, and the first 10 PCs.

Finally, we performed a meta-analysis of the model that was statistically significant in both the PAGE+ and KP cohorts (meta-analysis 1: *KIR2DL2* CN = 2, HLA-C1/C2, and adjusted for HLA-C*06:02 and HLA-C*12:03) as well as models that were statistically significant in only the KP cohort (meta-analysis 2: *KIR2DL2* CN = 1, HLA-C1/C2, and adjusted for HLA-C*06:02 and HLA-C*12:03) (**Figure 6**). For each meta-analysis, a fixed-effects model was implemented as there was no evidence of heterogeneity between the two cohorts *(p* > 0.05*).* With two copies of *KIR2DL2* (meta-analysis 1), the fixed-effects odds ratio was 1.33 [95% CI (1.13 – 1.57)] while the effect size was still in the same direction but lower with only one copy of *KIR2DL2* (meta-analysis 2), with a fixed-effects odds ratio of 1.13 [95% CI (1.03 – 1.23)].

## Discussion

### HLA and KIR Imputation Into GWAS Datasets

While several studies of the role of HLA and KIR have been conducted over the last several decades, most of these studies have been small-scale and likely underpowered and results have been inconsistent, for instance for *KIR2DS1* and for *KIR3DL1*. In this study, we have overcome the sample size limitations due to the relatively high cost of HLA and KIR genotyping by imputing HLA alleles and KIR CN into existing GWAS datasets, a strategy which we show can be cost-effective for groups wishing to investigate the role of HLA and KIR in other conditions, using large GWAS datasets such as the UK Biobank and other forthcoming databases that will be available to researchers. In addition to confirming that HLA-C2 as well as HLA-Bw4 are associated with psoriasis, we have shown that with respect to KIR CN, no KIR gene alone is associated with psoriasis. However, we have shown that in the presence of both its cognate ligands HLA-C1 and HLA-C2, *KIR2DL2* copy number is associated with psoriasis. At the time of this study, we are the first to report an association of *KIR2DL2* with psoriasis.

### KIR CN Alone Is Not Associated With Psoriasis

In this study, we observed that imputed KIR CN alone is not sufficient to affect psoriasis risk, not even previously implicated KIR genes such as *KIR3DL1* and *KIR2DS1*. It is possible that KIR *alleles* and *allotypes* are associated with psoriasis risk but KIR CN alone is not sufficient to account for psoriasis risk. Even more importantly, due to the lack of genetic linkage between KIR (chromosome 19) and HLA (chromosome 6), the effect of KIR CN might not be observed in the absence of its cognate HLA ligand. According to Moesta and Parham (35), many individuals with a given KIR gene may lack the cognate HLA class I ligand because co-evolution of KIR with HLA class I does not necessarily lead to pre-disposing combinations of HLA and KIR being inherited together.

### Two Copies of *KIR2DL2* in Individuals With HLA-C1/C2 Have Increased Risk for Psoriasis

While previous studies have implicated *KIR2DS1* as a significant factor in the pathogenesis of psoriasis, we did not observe a significant association between *KIR2DS1* copy number and psoriasis, either alone or in the presence of its HLA ligand, HLA-C2. However, when we stratified for HLA-C1/C2 heterozygous individuals, we observed a significant increase in risk for psoriasis for individuals harboring two copies of *KIR2DL2*, a result that remained true even after adjusting for the effect of the canonical psoriasis high-risk alleles, HLA-C*06:02 (a C2 allele) and HLA-C*12:03 (a C1 allele). In addition to replicating this finding in the KP cohort, we also observed that amongst individuals with HLA-C1/C2, having just one copy of *KIR2DL2* significantly increased risk of psoriasis [OR 1.17 (95% CI 1.03-1.33)], indicating a dose-response relationship. Finally, our meta-analysis of the PAGE+ and KP cohorts confirmed that there is an elevated risk for developing psoriasis in individuals that carry one or two copies of *KIR2DL2* and are HLA-C1/C2 heterozygotes.

There are several possible interpretations of our findings. Psoriasis is caused by a combination of factors involving the adaptive and innate immune system (45). With regards to the innate immune system, natural killer (NK) cells have been found to play a role in the regulation of immune-mediated and autoimmune disorders such as psoriasis. Several studies have shown reduced cell cytotoxicity and increased interferon-gamma production in NK cells from psoriatic patients compared to healthy controls (46–48). In the autoimmune disorder multiple sclerosis, NK cell depletion is associated with increased disease activity, suggesting that in certain autoimmune disorders NK cells may play an immunoregulatory role (49). While the mechanism of this immunoregulation by NK cells is not yet clear, it is likely that NK cell education plays an important role. The NK cell education model proposes that the threshold for NK cell activation and sensitivity to inhibition by self-MHC on autologous cells are on an allelic gradient. In this model, “uneducated” NK cells expressing inhibitory KIR without corresponding HLA ligands on autologous cells have the highest threshold for activation and the lowest sensitivity to inhibition by self-MHC, while “highly educated” NK cells that are homozygous for inhibitory KIR and the corresponding HLA ligands have the lowest threshold for activation and the highest sensitivity to inhibition by self-MHC (50). As such, the increase in psoriasis risk that we observed in the PAGE+ and KP cohorts for individuals with two copies of *KIR2DL2* and HLA-C1/C2 may be due to the NK cells in these individuals being in an intermediary education state whereby their activation threshold is just high enough to lead to a dysregulated state.

Compared to NK cells, it is more firmly established that T cells play a critical role in the initiation and maintenance of psoriasis. Recently, the role of CD8+ T cells has gathered attention (51–53), as these cells, along with their CD4+ T cell counterparts, secrete the pathogenic cytokine, IL-17. Interestingly, CD8+ T cells also express KIRs and it has been shown that CD8+ T cell survival after activation is partially dependent on expression of inhibitory KIRs. As noted by Seich al Basatena et al. (54), the inhibitory *KIR2DL2* may act by either directly regulating NK cell mediated immunity or by regulating T cell mediated immunity. A follow-up study by Boelen et al. (55) that included both in-vitro CD8+ T cell survival data and HLA-KIR genetic data concluded that inhibitory KIRs directly regulate T cell mediated immunity by increasing CD8+ T cell survival. While terminally differentiated CD8+ T cells stochastically express KIRs, Björkström et al. (56) have shown that most individuals exhibit a narrow repertoire of KIR typically dominated by one KIR gene. Interestingly, they also showed that the specificity of inhibitory KIR genes on CD8+ T cells is distinct from that of KIR genes expressed on NK cells from the same individual, suggesting the possibility that a dominant KIR gene (i.e. *KIR2DL2*) expressed on the surface of CD8+ T cells may not be expressed on NK cells from the same individual. Given that the studies over the last decade have provided much more consensus on the direct role of CD8+ T cells on psoriasis initiation and maintenance (9, 51) than NK cells (48, 57), it is possible that carriers of *KIR2DL2* are at greater risk for developing psoriasis because those individuals are more likely to have enhanced survival of skin resident memory CD8+ T cells that can be activated by dendritic cells and release cytokines such as IL-17.

However, it is not entirely clear at present whether having two copies of *KIR2DL2* and having HLA-C1/C2 principally drive NK cell dysfunction due to incomplete NK cell education, increased survival of potentially pathogenic CD8+ T cells, or both mechanisms acting in concert to increase risk of developing psoriasis. Future in-vitro studies of NK cell dysfunction and CD8+ T cell survival from individuals with psoriasis that are homozygous for inhibitory KIR and heterozygous for the respective HLA ligands may shed more light. Finally, as *KIR2DS2* is in near complete linkage disequilibrium with *KIR2DL2*, we cannot rule out the possibility that the observed association between *KIR2DL2* CN and psoriasis may in part be due to the presence of *KIR2DS2*. However, it should be noted that HLA-C1 and HLA-C2 are not thought to be ligands for *KIR2DS2* (58). Our future studies of KIR CN may also investigate the role of *KIR2DS2.*

### *KIR3DL1* and HLA-Bw4-80I

In a previous small-scale study (14), we showed that the *low* and *null* allotypes of *KIR3DL1* are associated with increased and decreased risk for psoriasis, respectively. We also showed that *KIR3DL1 low* in combination with HLA-Bw4 significantly increases risk for psoriasis. In the present study we discovered that *KIR3DL1* copy number alone is not significantly associated with psoriasis while HLA-Bw4 and HLA-Bw4-80I were both significantly associated with psoriasis, even after adjusting for the known high-risk allele HLA-C*06:02. Furthermore, we observed that KIR3DL1 copy number in models stratified on HLA-Bw4 or HLA-Bw4-80I were not significantly associated with psoriasis. It is possible that a significant association was not observed because *KIR3DL1* copy number does not account for whether cell surface expression of *KIR3DL1* will be *low*, *high*, or *null*. Our future efforts include developing an update to KIR*IMP to impute not only KIR CN, but also KIR alleles. Imputation of KIR alleles will allow us to develop models that include allotypes of *KIR3DL1* (or any other KIR gene that we can successfully impute).

### Limitations

While this present work has only tested imputed KIR CN for association with psoriasis, we hope to test imputed KIR alleles in the near future after updates to the current version of KIR*IMP are implemented. Testing imputed KIR alleles will allow for replication of previous KIR allelic associations, such as the protective effect of *KIR3DL1* Null (14, 15). While this present study includes samples from individuals of European descent, future studies may also include samples from individuals of African American and East Asian descent.

### Conclusions

While previous studies of the role of KIR genes have been limited by small sample sizes, we have implemented state-of-the-art imputation methods to impute KIR CN and HLA alleles into thousands of samples from previous GWAS. Our results show that HLA-C1/C2 heterozygous carriers with 2 copies of *KIR2DL2* are at an elevated risk for developing psoriasis.

## Data Availability Statement

The datasets presented in this study can be found in online repositories. The names of the repository/repositories and accession number(s) can be found below: (https://doi.org/10.6084/m9.figshare.14132375.v1).

## Ethics Statement

The studies involving human participants were reviewed and approved by UCSF Human Research Protection Program. The patients/participants provided their written informed consent to participate in this study.

## Author Contributions

WiL, RA, and SL designed the study. RA and WL drafted the manuscript. WiL, SL, and DV provided critical revisions of the manuscript. RA performed statistical analysis of imputed data. DV, AM, and DS performed KIR and HLA imputation. JN, EE, RN, and LT performed preparation of GWAS data. EJ, JE, AF, WiL, SW, JO, JF, and WiL provided GWAS datasets. JH and PN performed NGS KIR typing. All authors contributed to the article and approved the submitted version.

## Funding

This study was supported by an NIH grant to WL (U01 AI119125) and a UCSF/Kaiser Permanente Division of Research Grant for Fellows to RA (RNG 021377). RA was also supported by a NIAMS postdoctoral training grant to the Department of Dermatology at the University of California, San Francisco (T32 AR007175-38). The funding bodies played no role in the collection, analysis, or interpretation of data in this study.

## Conflict of Interest

WL has received research grant funding from Abbvie, Amgen, Janssen, Leo, Novartis, Pfizer, Regeneron, and TRex Bio.

The remaining authors declare that the research was conducted in the absence of any commercial or financial relationships that could be construed as a potential conflict of interest.
