# Supplementary material for: Large-Scale Imputation of KIR Copy Number and HLA Alleles in North American and European Psoriasis Case-Control Cohorts Reveals Association of Inhibitory KIR2DL2 With Psoriasis
Source: Front Immunol. 2021 Jun 11;12:684326. doi: 10.3389/fimmu.2021.684326 (PMC8231283; doi:10.3389/fimmu.2021.684326)
Supplement: Supplementary file 1 [file DataSheet_1.docx]

**SUPPLEMENTARY METHODS**

**Additional details on genotyping of UCSF and UD samples**

We used recommended parameters in the Affymetrix Best Practices Workflow [Affymetrix, “Axiom® Genotyping Solution Data Analysis Guide”, 2014. <http://media.affymetrix.com/support/downloads/manuals/axiom_genotyping_solution_analysis_guide.pdf>]. This workflow includes rejecting samples with DishQC < 0.82 and samples with a first-pass sample call rate < 0.97. After this first round of QC, we implemented another round of genotype QC by excluding SNPs with a call rate below 0.95 across all samples or that had a Hardy-Weinberg Equilibrium (HWE) p-value < 1x10^-6^ (21,421 SNPs were excluded). Samples with call rates across all SNPs below 0.98, duplicates (π_hat > 0.98), and first-degree relatives (0.4 < π_hat < 0.6) were excluded (110 samples were excluded).

**Additional details for principal components analysis (PCA)**

We used EIGENSTRAT to perform principal component analysis with the following parameters:

- Iterations = 3
- Number of eigenvectors to be used = 3
- Sigma threshold = 5

*QC of UCSF and UD datasets*

Before performing PCA, we applied the following QC procedures:

- Filtered SNPs with greater than 5% missing genotypes —> 0 SNPs dropped
- Filtered individuals with greater than 5% missing SNP data —> 0 individuals dropped
- Kept only SNPs (n = 724,470) that were common to all three batches of genotyping
- Kept only individuals that self-reported as being of European descent
- Performed IBD analysis to identify duplicates and first-degree relatives —> 110 individuals dropped
- Performed HWE analysis in control samples —> 21,421 SNPs dropped

*QC of PAGE consortium “null” SNPs*

In addition to using chromosomes 6 and 19 from the PAGE consortium dataset to impute HLA alleles and KIR copy number genes, we also used a set of “null” SNPs (n = 9,708) from the PAGE consortium dataset that the Elder lab had previously identified as having no association with psoriasis. After removing 749 CG/AT SNPs, there were 8,959 SNPs in 3,580 cases and 5,902 controls.

*Merging of UCSF, NF, and PAGE consortium datasets*

There were 1,753 SNPs in common between the UCSF, UD, and PAGE consortium datasets after merging. We dropped 20 SNPs in areas of known long-range LD and dropped 1 SNP after LD-pruning, leaving us with 1,732 SNPs for PCA.

**Fig. 1** Scree plot of the PCA to determine how many PCs to use to adjust for sub-population stratification.

**Fig. 2** A PCA plot showing the first two PCs, with cases in blue and controls in red.

**Fig. 3** A PCA plot showing PC 1 against PC3, with cases in blue and controls in red.

**Additional details on KIR and HLA imputation**

*QC and pre-phasing*

SNP genotypes in windows centred on the HLA (chromosome 6, 25-35 Mb) and KIR (chromosome 19, 53-58 Mb) regions were extracted.

Individuals and SNPs with > 10% missingness were removed. This resulted in the removal of no individuals.

*Kaiser Permanente*

The Kaiser Permanente RPGEH data set was converted from GRCh36 to GRCH37 coordinates using UCSC LiftOver. All other data sets were already in GRCh37 coordinates.

Phased haplotypes were computed for each data set, as required as input by the imputation methods. SHAPEIT v2 was used to phase all data sets except for the Kaiser Permanente RPGEH European data. The '--duohmm' flag was used to fully utilize pedigree information where available. For the Kaiser Permanted RPGEH European data, SHAPEIT v3 was used, as recommended for large data sets with > 20,000 samples (this data set had 66,000 samples) using the recommended '--fast' setting. 1000 Genomes Project Phase 3 data was used as the reference panel for all phasing.

The directly genotyped SNP data was used to perform KIR imputation. Accuracy estimates obtained from KIR*IMP indicated lower than expected accuracy due to the number of SNPs overlapping with the reference panel. For this reason SNPs were imputed in each of the cohorts. This was done by uploading the SNP data to the Michigan Imputation Server (https://imputationserver.sph.umich.edu) which performs phasing with Minimac3.

The imputed SNP data was used to perform KIR imputation. We only used high quality imputed SNPs with R-squared > 0.8. We also discarded imputed SNPs with an allele frequency that differed by > 0.15 from the SNP allele frequency in the KIR reference panel. This ensured that the imputed SNPs used for KIR imputation were of high quality. We also verified concordance between the two sets of KIR imputations obtained with directly genotyped and imputed SNPs. (Imputations with high posteriors should be the same in both sets of KIR imputations.)
